# Accidents at work among employed persons in Germany: Results from the 2024 ‘Health in Germany’ panel

**DOI:** 10.25646/14402

**Published:** 2026-09-23

**Authors:** Alexander Rommel, Ronny Kuhnert, Anke-Christine Saß

**Affiliations:** Robert Koch Institute, Department of Epidemiology and Health Monitoring, Berlin, Germany

**Keywords:** Work-related accidents, Work-related injuries, Accidents, Accident prevention, Workplace, Education, Germany

## Abstract

**Background:**

Accidents at work can result in injuries, health care utilisation, incapacity to work and long-term consequences. Population surveys complement statistics on notifiable work-related accidents, among other things by also capturing less severe accidents at work.

**Methods:**

The authors analysed data from the 2024 ‘Health in Germany’ panel. Restricted to the most recent unintentional injury requiring medical treatment, the indicator describes the occurrence of an accident at work among employed persons aged 18 to 70 years.

**Results:**

Based on the indicator, ‘most recent unintentional injury’, 2.4 % of employed persons experience an accident at work within a 12-month period. Men are more affected than women. Accidents at work are more common among younger men and men with low education.

**Conclusions:**

Population surveys also capture minor unintentional injuries and can identify important starting points for injury prevention. Efforts to prevent accidents at work should focus particularly on occupations frequently performed by younger men and socioeconomically disadvantaged people, such as manual and low-skilled service occupations.

**Key messages**

► For 2.4 % of employed persons 70 years old and younger, the most recent accident in the previous 12 months was an accident at work. Extrapolated to the population, this corresponds to approximately 885,000 people affected per year.
► Accidents at work affect women (1.5 %) less frequently than men (3.2 %).
► Accidents at work are particularly common among younger men and men with low education.

**The 2024 RKI ‘Health in Germany’ panel**

**Data holder:** Robert Koch Institute

**Objectives:** To provide comprehensive data on the health status, health-related behaviour and health care of the population in Germany and to enable future longitudinal comparisons and analysis of trends over time

**Study design:** Panel study with a mixed-mode approach (participation online or by postal questionnaire)

**Population:** German-speaking population aged 18 and over who live in private households and have their main residence in Germany

**Sample:** Probabilistic/representative sample of the ‘Health in Germany’ panel infrastructure

**Participants in the** **2024** **annual wave:** A total of 41,376 of the individuals registered in the panel took part in at least one of the three sub-waves in 2024.

Questionnaire A: 14,759 women, 12,374 men,

66 persons with other gender identities

Questionnaire B: 14,828 women, 12,258 men,

61 persons with other gender identities

Questionnaire C: 14,709 women, 12,329 men,

64 persons with other gender identities

Questionnaire D: 14,872 women, 12,368 men,

66 persons with other gender identities

**Data collection:**

1st sub-wave: 28.05.2024 – 05.08.2024

2nd sub-wave: 12.08.2024 –14.10.2024

3rd sub-wave: 28.10.2024 – 06.01.2025

More information at

## 1. Introduction

Most injuries occur unintentionally as a result of accidents [1]. In 2024, around 7 million people in Germany sustained unintentional injuries that required medical treatment [2]. More than 34,000 people died as a result of unintentional injuries [3]. Around 5 % of total healthcare expenditure in Germany is spent on the medical treatment of injuries (International Classification of Diseases, 10th Revision (ICD-10): S00 – T98) [4]. In addition to immediate treatment costs, unintentional injuries can result in incapacity for work, longer-term health consequences and limitations in the capacity to work. However, official statistics provide only a partial picture of unintentional injuries [5]. In 2024, 810,399 work-related accidents were officially notified, with a downward trend [6]. However, accidents at work are only required to be reported if they result in more than three days of incapacity for work or in death. Less severe accidents at work are not notifiable but are only documented internally by employers [7]. Survey data are therefore an important complement for estimating population-based prevalences of non-fatal accidents at work and describing sociodemographic differences.

## 2. Methods

### Study design and sample

The Robert Koch Institute (RKI) established the ‘Health in Germany’ panel in 2024 as part of a recruitment study. The sampling was based on a double-stratified random selection: 359 primary sampling units, known as sample points, were randomly drawn from all municipalities in Germany, taking into account the federal state and BIK municipality size class [8] (first selection stage). In the second selection stage, addresses were drawn for each sample point from the address registers of the respective residents’ registration offices, with stratification by age group. The selected individuals were invited to participate in a short survey and asked for their consent to participate in future surveys as part of the panel [9]. 29 % of those invited registered for the panel. A mixed-mode approach was used, which enabled both online participation (Computer-Assisted Web Interview, CAWI) and written-postal participation (Paper-and-Pencil Interview, PAPI) [10]. For its first annual wave in 2024, the RKI panel comprised 46,863 registered participants aged 18 and over: 24,881 women, 21,856 men and 126 people with other gender identities. The response rate (the proportion of participants in relation to the people registered in the panel), calculated in accordance with the standards of the American Association for Public Opinion Research (AAPOR [11]), ranged from 75 % to 81 % across the individual sub-waves. A detailed description of the methodology and response rate (including stratification by age and gender) can be found elsewhere [12].

### Variables

To describe gender differences, the authors used participants’ own specification of their gender identity. The analyses by gender included people who identified as female or male. The analyses included gender-diverse people in the overall category, meaning that the total number of cases differed from the sum of male and female cases. Education-level was classified according to the Comparative Analysis of Social Mobility in Industrial Nations’ (CASMIN) concept [13], based on participants’ reported highest educational and vocational qualifications. The different classifications for educational level were low, medium or high.

To describe the epidemiology of unintentional injuries in the population, the 2024 RKI panel recorded the prevalence of injuries during the 12 months preceding the survey (only unintentional injuries followed by medical treatment). The survey requested detailed information on each participant’s most recent unintentional injury. Each person who had experienced an unintentional injury received 20 specific questions on the place and mechanism of the accident, type of injuries, care received and any subsequent incapacity for work. The key distinguishing characteristic of unintentional injuries is the place of accident, i.e. the setting in which an unintentional injury occurs (work, home, leisure, road traffic). As the focus here is specifically on accidents at the workplace and during occupational activities, the analyses below exclude unintentional injuries in educational establishments and commuting accidents, consistent with previous analyses [14, 15]. Thus, based on the most recent unintentional injury requiring medical treatment, the indicator describes the occurrence of an accident at work among employed persons aged 18 to 70 years. Again, based on the most recent unintentional injury requiring medical treatment, an additional indicator presents the proportion of accidents at work among all reported unintentional injuries in the total population.

### Weighting

In order to correct as far as possible for distortions due to selective participation and discrepancies between the composition of the sample and that of the population, the authors calculated a multi-stage sample weight [16]. In the initial recruitment study, the weighting accounted for the sample design and an adjustment against population figures as of December 31, 2023, and the 2021 Microcensus. The following factors were taken into account: age, gender, federal state, BIK municipality size class [8], education groups based on CASMIN [13] and household size (single-person vs. multi-person household). The exact procedure is described elsewhere [16].

The analyses are based on the 2024 annual survey of the RKI panel, dataset version 6, and were conducted using the statistical software R, version 4.5.1.

## 3. Results

Overall, 2.4 % of employed persons 70 years old and younger reported that their most recent unintentional injury in the previous 12 months was an accident at work. Thus, among most recent unintentional injuries, accidents at work accounted for 15.1 % of all unintentional injuries in the adult population. Extrapolated to the population, this corresponds to approximately 885,000 people affected in 2024. Women in employment were less frequently affected by accidents at work (1.5 %) than men (3.2 %). Accidents at work accounted for 9.6 % of unintentional injuries among women and 20.1 % among men. There was no clear age pattern among women. Among men, the higher prevalence of 5.3 % in the 18 – 29-year age group was notable (Table 1, Supplementary material Table A1).

**Figure 1:**
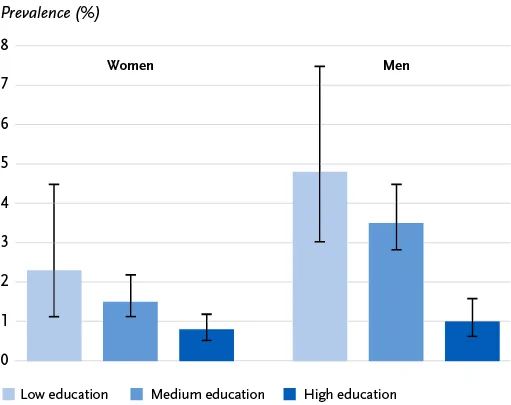
Prevalence of accidents at work (most recent accident) by education and gender (n = 7,416 employed women, n = 6,214 employed men, n = 29 employed gender-diverse persons). Source: 2024 RKI panel

Marked differences in accidents at work were also found between education groups. Prevalence, measured on the basis of the most recently reported unintentional injury, was comparatively high particularly among employed men with low education (4.8 %), followed by men with medium (3.5 %) and high education (1.0 %). Among men with low education, 27.0 % reported that their most recent unintentional injury occurred at work. Among women with low (2.3 %) and medium education (1.5 %), prevalence was only around half as high as among men in the corresponding education group. The prevalence of accidents at work among employed women in the high education group (0.8 %) differed little from that among men in the high education group. Differences between education groups were therefore less pronounced among women than among men (Figure 1).

## 4. Discussion

Among employed persons 70 years old and younger, 2.4 % had experienced an accident at work as their most recent unintentional injury in the previous 12 months. Men were generally more affected than women. Accidents at work were comparatively common among young men and people with low education. These associations are known from previous analyses [14, 15]. One reason why men are more affected is that they are considerably more likely than women to work in accident-prone occupations and activities [5]. According to data from the German Social Accident Insurance, men account for 72.7 % of all notifiable work-related accidents and around 94.5 % of all fatal accidents at work [17]. Limited occupational experience means that young age is itself an independent risk factor, particularly for men [15]. Socioeconomically disadvantaged employed persons likewise face a higher risk at work because low education is disproportionately associated with accident-prone occupations and occupational activities. These generally include manual occupations as well as low-skilled service occupations, for example in manufacturing or construction, but also in health and social care [6, 15, 17].

The extrapolated estimate from the 2024 panel of around 885,000 employed persons who experienced an accident at work is higher than the officially reported 810,000 work-related accidents for 2024 [6]. The panel also captures accidents at work that are not notifiable because the injuries are less severe. Viewed in this way, the two data sources are of a similar order of magnitude. At the same time, the present estimate should be regarded as conservative (see below). If detailed information were available for all accidents in the previous 12 months, the estimate would be somewhat higher.

The temporal trend in the survey data should also be assessed in this light. As in the official statistics, RKI survey data suggest at first glance that the extrapolated number of accidents at work has declined over the past 15 years. Earlier analyses of the 2010 German Health Update study found a prevalence of 2.8 % and, when extrapolated, slightly more than 1 million accidents at work [14, 15]. However, respondents in 2010 could report up to three unintentional injuries per year, so no clear trend can be inferred.

### Strengths and limitations

A key strength is the large number of participants in the ‘Health in Germany’ panel. Recruitment through residents’ registration offices ensures a high degree of representativeness for the adult resident population in Germany. The analyses also apply a study-specific weighting factor to correct for different probabilities of non-response. Nevertheless, certain biases, such as selective non-participation (selection bias), cannot be completely ruled out. People who are interested in health issues and those with more health-conscious behaviour are more likely to participate. A further limitation is that participation requires good written German skills.

With regard to the assessment of accidents at work, a limitation is that the 2024 panel collected detailed information only for the most recent unintentional injury. Almost one third of people who experienced an unintentional injury reported multiple unintentional injuries within 12 months (women 27.2 %, men 30.3 %) [2]. In this respect, the present analyses provide a conservative estimate of the frequency of accidents at work. A further limitation of the present analysis is the absence of occupational coding in the 2024 panel. Occupation and work activity are the strongest predictors of an accident at work [15]. The highest educational qualification examined here provides only a rough approximation.

### Conclusion

In conclusion, there remains considerable potential for prevention in relation to accidents at work, which accident insurance funds and other occupational health and safety stakeholders continuously address [e.g. 6]. For example, the frequency of notifiable work-related accidents has declined most markedly among young employees [17]. Nevertheless, in light of survey data, the prevalence of accidents at work appears to be relatively stable, as less severe work-related accidents are not necessarily notifiable. Prevention approaches should identify and specifically address risk groups, with particular attention to high-risk activities that are especially often performed by younger men [18]. As minor accidents must also be documented internally if first aid was provided [7], they offer an important starting point for preventing more serious events.

## Figures and Tables

**Table 1:** Prevalence of accidents at work (most recent unintentional injury) (n = 7,416 employed women, n = 6,214 employed men, n = 29 employed gender-diverse persons) and proportion of accidents at work among all unintentional injuries (most recent accident) (n = 1,361 women who experienced an unintentional injury, n = 1,238 men who experienced an unintentional injury, n = 17 gender-diverse persons who experienced an unintentional injury), by gender and age. Source: 2024 RKI panel

**Gender**	**Age**	**Prevalence %**	**(95 % CI)**	**Proportion among all places of accident %**	**(95 % CI)**
Overall	Overall	2.4	(2.0 – 2.9)	15.1	(13.1 –17.4)
Women	Overall	1.5	(1.1 – 2.0)	9.6	(7.5 –12.2)
	18 – 29 years	1.4	(0.5 – 3.4)	10.2	(5.7 –17.6)
	30 – 44 years	1.7	(1.0 – 2.8)	14.3	(8.9 – 22.1)
	45 – 64 years	1.4	(0.9 – 2.0)	14.1	(9.7 – 20.1)
	65 – 70 years	2.0	(0.5 – 8.0)	0.8	(0.3 – 2.1)
Men	Overall	3.2	(2.6 – 4.0)	20.1	(17.0 – 23.7)
	18 – 29 years	5.3	(3.4 – 8.4)	23.4	(16.2 – 32.5)
	30 – 44 years	2.3	(1.6 – 3.5)	20.1	(14.2 – 27.6)
	45 – 64 years	3.2	(2.4 – 4.2)	27.5	(21.8 – 34.0)
	65 – 70 years	3.2	(0.8 –12.2)	3.8	(1.8 – 7.8)

CI = confidence interval
